# No Medication for My Child! A Naturalistic Study on the Treatment Preferences for and Effects of Cogmed Working Memory Training Versus Psychostimulant Medication in Clinically Referred Youth with ADHD

**DOI:** 10.1007/s10578-018-0812-x

**Published:** 2018-05-16

**Authors:** Peter Muris, Dorien Roodenrijs, Lut Kelgtermans, Sonja Sliwinski, Ulrike Berlage, Hanna Baillieux, Anne Deckers, Marieke Gunther, Bertien Paanakker, Ida Holterman

**Affiliations:** 1 Department of Clinical Psychological Science, Maastricht University, P.O. Box 616, 6200 MD Maastricht, The Netherlands; 2 Lucertis (Virenze-RIAGG) Maastricht, Maastricht, The Netherlands; 3 Stellenbosch University, Stellenbosch, South Africa

**Keywords:** Attention-deficit/hyperactivity disorder, Cogmed working memory training, Stimulant medication, Treatment preferences, Clinical effectiveness, Naturalistic clinical study

## Abstract

In this naturalistic clinical study, we explored the applicability and clinical effectiveness of Cogmed WMT, pharmacotherapy, and their combination for clinically referred children and adolescents with attention-deficit/hyperactivity disorder (ADHD). Ninety youth with ADHD (ages 6–16 years) and their parents were offered the possibility to choose one of the three interventions. The motives for choosing various interventions were quite different. Medication was chosen because this treatment was expected to be most effective, but also because the Cogmed WMT program was regarded as too taxing. The choice for Cogmed WMT was mainly negatively motivated: participants tended to be strongly against the use of medication, found it a too rigorous step, or feared side effects and addiction problems. The choice for the combination treatment was strongly positively motivated: parents and youth indicated that they wanted to receive the best possible intervention and part of them also had high expectations of Cogmed WMT. In terms of clinical effectiveness, pharmacotherapy with stimulant medication and the combination treatment produced larger reductions in ADHD symptomatology than Cogmed WMT. Further, results indicated that Cogmed WMT selectively enhanced working memory performance. Finally, after conducting Cogmed WMT, youths and parents were more ‘open’ to accept pharmacotherapy as intervention, probably because the training increased greater insight in and awareness of the problematic features of ADHD.

## Introduction

Attention-Deficit/Hyperactivity Disorder (ADHD) is characterized by symptoms of extreme inattention, hyperactivity, and impulsivity that interfere significantly with daily functioning and development [1, 2]. About 7% of the children and adolescents meet the diagnostic criteria of this disorder at some point before the age of 18 [3], and these include 3 to 9 times as much boys than girls [4]. Because ADHD is a persistent problem—with symptoms often continuing to last into adulthood [5]—and is associated with diminished performance at school, social rejection, and the development of disruptive behavior problems [6], it is not surprising that parents often seek professional help for their child. In the United States, around 30% of all referrals to mental health services involve children and adolescents with this disorder [7] and a highly similar percentage has been documented in The Netherlands, where the current study was carried out [8].

There is no curative intervention for ADHD, although good care and treatment may help to normalize the behavior of these youngsters and to improve their eventual prognosis. Pharmacotherapy with psychostimulant medication such as methylphenidate and amphetamine is considered to be the first-line treatment [9, 10]. Placebo-controlled research has clearly demonstrated that that this type of medication is effective: in about 70% of the children and adolescents the intensity of the prototypical symptoms of the disorder is substantially reduced [11], a treatment effect that is associated with a large effect size of around 1 [12]. The positive effects of psychostimulant medication have been shown to be retained over longer time periods [13] and to eventually reduce the risk for developing comorbid psychiatric disorders [14] and academic difficulties [15].

In spite of these favorable outcomes, a considerable proportion of the parents dismiss psychostimulant medication as an intervention for their youngster with ADHD [16], and there are a number of explanations for this refusal. The first one pertains to parents’ acceptance of the diagnosis ADHD for their child. In some youth, the extensive assessment consisting of a developmental anamnesis, interviews with child, parent(s), and teacher(s), behavioral observations, questionnaires, and performance-based (neuropsychological) attentional tests yield a clear-cut picture that leaves little doubt about the diagnosis of the child. Most of the times, however, the assessment results are less consistent. For example, parents are often less aware of the child’s behavior during scholastic, task-oriented activities that strongly call upon cognitive processes such as self-control and concentration [17]. In these cases, the chance increases that parents do not (fully) recognize the problem of their child [18], which in turn undermines their willingness to accept a pharmacological intervention with psychostimulants. Another reason is concerned with a negative attitude of parents regarding the prescription of medication for children and adolescents with ADHD. This attitude can be partly based on information provided by the media: a popular message in the lay press is that ADHD is an over-diagnosed condition in mental health care, and that as a consequence too many young people are unjustly treated with psychostimulant medication. For instance, an investigation by Dosreis et al. [19] has shown that 55% of the parents because of this reason are reluctant to accept pharmacological treatment for their child with ADHD. Other parents are skeptical about the positive long-term effects of psychostimulants (30%), are convinced that this type of medication will have serious side-effects (14%) or increase the risk of substance use problems (8%). Although there is no empirical support for these misconceptions, there is no doubt that parents act on them when choosing an intervention for their child with ADHD. For example, Sciutto [20] noted that the stronger parents belief in faulty ideas about medication, the less likely they are in accepting pharmacotherapy with psychostimulants as an intervention for ADHD.

In search for a good alternative treatment for children and adolescents with ADHD, parents could opt for behavior therapy. This intervention has also been shown to yield favorable results, although some meta-analyses have indicated that the effects of this psychosocial intervention are smaller than those produced by pharmacotherapy with methylphenidate, the most commonly prescribed psychostimulant drug [21]. Working memory training is another non-pharmacological intervention that has been put forward as a viable treatment option for youth with ADHD. Working memory refers to the cognitive system that is responsible for temporarily holding information available for further processing, which makes it possible to reflect upon one’s (possible) responses during an activity so that behavior is not dominated by the immediate sensory input from the environment [22]. Barkley [23] was among the first scholars to assume that the prototypical symptoms of inattention, hyperactivity, and impulsivity are (at least in part) the result of a dysfunctional working memory, and indeed a meta-analytic review by Martinussen et al. [22] demonstrated that youth with ADHD display significant impairments in this cognitive system. Based on this notion the idea was born that symptoms of ADHD can be alleviated by a cognitive training for improving working memory.

With this in mind, Klingberg et al. [24] developed their Cogmed working memory training (WMT), which consists of a series of visuospatial (e.g., remembering the position of objects in a complex grid) as well as verbal exercises (e.g., remembering letters and digits) that have to be conducted on a computer at home for 1 h per day, 5 days per week, 5 weeks long. In a first controlled trial [25], 53 seven- to 12-year-old children were randomly allocated to either Cogmed WMT or a control condition (in which exercises were so easy that no training of working memory occurred). Results indicated that the Cogmed WMT yielded positive effects, which were not present in the control condition. First of all, the results showed that children’s working memory performance clearly improved as a result of the training. Further and most importantly, the parents of the children in the WMT condition reported that their offspring also displayed a significant decrease in symptoms of inattention, hyperactivity, and impulsivity. The positive finding regarding the improvement in working memory following Cogmed WMT has been replicated in various studies [26], but the evidence showing that such a training program also produces a significant decrease in ADHD symptomatology is more mixed [27–30].

Furthermore, while research on the efficacy of Cogmed WMT is important in order to learn more about performance of this intervention under ideal, well-controlled circumstances, studies are also needed on effectiveness, thus its performance under real-life conditions. The present investigation was conducted to examine the applicability and effects of Cogmed WMT (as compared to pharmacotherapy) in an everyday clinical practice. Ninety consecutively referred children and adolescents who were diagnosed with ADHD at our outpatient treatment center participated in this naturalistic trial. Youth and parents were given information about three available interventions: (1) Cogmed WMT, (2) pharmacotherapy with psychostimulant medication, and (3) a combination of Cogmed WMT and pharmacotherapy, after which they were invited to choose one of these treatment options. First of all, we wanted to learn more about the choice for various treatment options for children and adolescents with ADHD: what is the percentage of youth and parents selecting Cogmed WMT and/or pharmacotherapy as intervention(s) and what are the motives for their choice? Further, to evaluate the effects of the three interventions, pre- and post-intervention assessments were conducted that consisted of parent-, teacher-, and self-report rating scales measuring ADHD symptomatology, comorbid problems, and executive functioning, as well as performance-based tests for assessing working memory and attentional processes. Pre- to post-comparisons of parent- and teacher-ratings of ADHD symptoms were considered as the primary outcome measure [31], and were employed to get an impression of the effect sizes produced by various interventions. Finally, 1-year after the interventions had taken place, parents were contacted by telephone with follow-up questions on how they evaluated the chosen treatment in retrospect, the current symptom level of their child, and eventually other ADHD treatments that were/had been applied.

## Method

### Procedure and Participants

The intervention part of the study was conducted between November 2012 and June 2015 at Lucertis (previously Virenze-RIAGG) Maastricht, an outpatient center for children and adolescents with mental health problems. Youth who had been referred to the center were subjected to a standard intake procedure, which consisted of unstructured clinical interviews with the youngster and his/her parents, a semi-structured diagnostic (DSM-IV-based) interview, an observation in the play room (for children in the preschool and primary school age), a contact with the teacher at school, administration of a developmental anamnesis survey and a set of standardized questionnaires (i.e., the Achenbach Scales of Empirically-Based Assessment [32]), and eventually a (neuro)psychological test battery consisting of for example an intelligence test (in most cases the Wechsler Intelligence Scale for Children [33]) or a performance-based attention test (e.g., Test of Everyday Attention for Children (TEA-Ch) [34]). After the intake, a multidisciplinary team consisting of psychologists and other mental health workers, and at least one psychiatrist used all the gathered information to make a diagnosis and a DSM-IV-based classification for the child as well as to formulate a treatment plan.

Cases for which ADHD was identified as a condition that required treatment (*N* = 97) were approached for participation in the present study. This was done during an advisory conducted by the case manager (the psychologist who carried out the unstructured clinical interviews with youngster and parents) who (1) discussed the diagnosis and classification of ADHD, (2) explained why the team thought that the youngster suffered from this condition, and (3) provided basic psycho-education about the disorder. Following this, the youngster and parents were given a leaflet containing information about the background and rationale of the three interventions, their content, and possible disadvantages. The case manager discussed the content of the leaflet orally and also explained that there would be pre- and post-intervention assessment sessions for both the youngster (1 h per session) and the parents (20 min per session) to quantify the effects of the intervention. Then the youngster and the parents were given two weeks to decide on participation and the choice of the intervention.

Out of the 97 eligible participants, it appeared that five were already or had been taking psychostimulant medication. Because these participants were no longer naïve to one of the interventions, they were discarded from the study. Two further participants refused to participate because they did not want to participate in the extra assessment sessions. Thus, eventually 90 youngsters and their parents agreed to participate by signing the informed consent form, on which they also indicated the selected intervention, the reason(s) for this choice, and the level of confidence in the intervention as being helpful for their child (1 = not helpful at all, 10 = extremely helpful). The characteristics of the 90 participating youngsters are displayed in Table 1. The total sample consisted of 58 boys and 32 girls, who had a mean age of 129.59 months (10.80 years). The majority of participants were children aged 6–12 years (i.e., 71.1%) of whom most were in a regular primary school; others were adolescents aged 13 to 16 years (28.9%) who typically received education in secondary schools with varying levels. The mean IQ score of the participants in this sample was 103.13 (range: 80–144). With regard to the families: these frequently had a low to medium socio-economic background (84.0%) and in about one-third of the cases parents were divorced. Most youth had a father and a mother with an original Dutch origin; others had at least one parent coming from another country (e.g., Germany, Indonesia, Turkey, Morocco). All youth had a diagnosis of ADHD combined type, ADHD predominantly inattentive type, or ADHD-NOS and note that many of them also suffered from comorbid psychiatric conditions (e.g., parent–child relational problem, pervasive developmental disorder, relational problem due to a mental disorder), which was not an exclusion criterion.

**Table 1:** Demographic characteristics of the 90 youth participating in the present study

	Percentage, *n*, or *M* (*SD*)
Gender (boys/girls)	
Boys	64.4
Girls	35.6
Age (months)	129.59 (30.21)
6–12 years	71.1
13–16 years	28.9
Intelligence (IQ)^a^	103.13 (15.75)
Education	
Regular primary school	65.5
Special primary school	3.3
Lower vocational education	8.9
Higher general secondary education	7.8
Pre-university education	2.2
Special secondary school	1.1
International school	1.1
Nationality of parents	
Dutch	77.8
Other	22.2
Family structure	
Complete	65.4
Broken	34.6
Socio-economic status	
Low	45.7
Medium	38.3
High	16.0
ADHD diagnosis	
ADHD combined type	55.4
ADHD predominantly inattentive type	44.5
ADHD-NOS	1.1
Level of interference (ADIS)	1.1
Comorbidity	
Yes	61.7
No	38.3
Comorbid disorders^b^	
Parent–child relational problem	25
Pervasive developmental disorder	9
Relational problem related to a mental disorder	9
Dyslexia	7
ODD/disruptive behavior disorder NOS	7
Anxiety/mood disorder	6
Identity problem	5
Reactive attachment disorder	2
Sibling relational problem	2
Partner relational problem	1
Adjustment disorder	1
Enuresis	1

*ADIS* anxiety disorder interview schedule, ADHD section

^a^Only available for 55 participants

^b^Some youth had more than one comorbid diagnosis

**Fig. 1:**
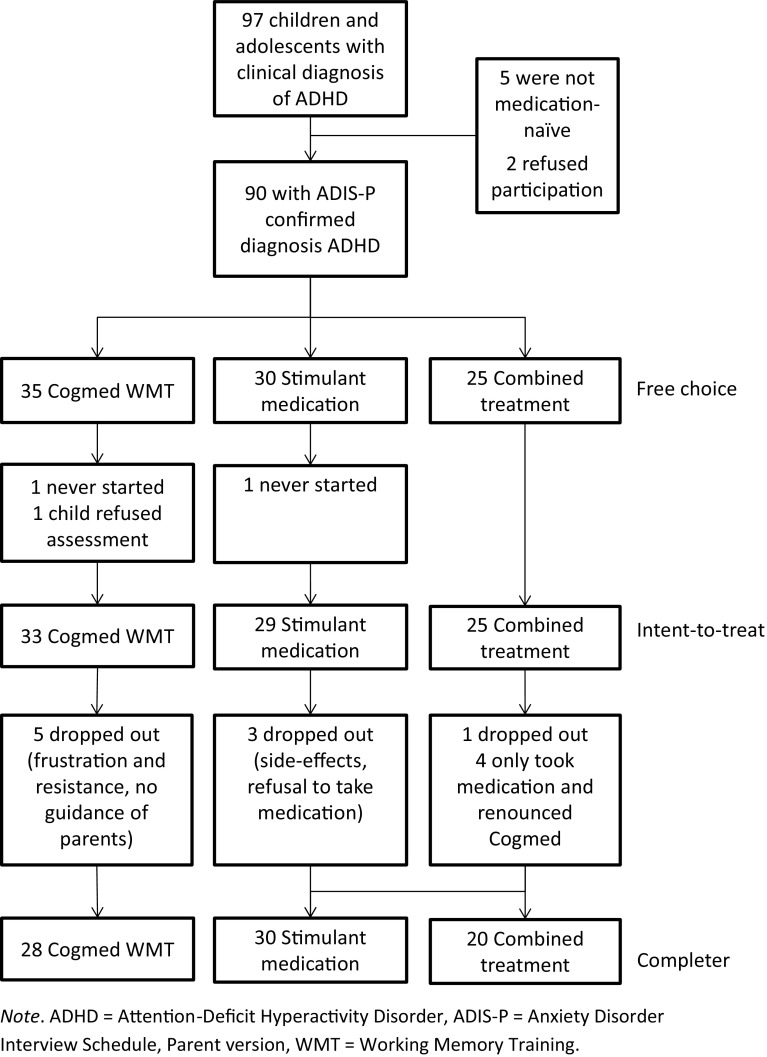
Flowchart of participants during various stages of this clinical study

As can be seen in the flowchart presented in Fig. 1, for 35 youngsters and their parents (38.9%) Cogmed WMT was the treatment of choice, 30 (33.3%) preferred the stimulant medication, and 25 (27.8%) chose the combination of WMT and medication as intervention. The three groups differed with regard to a number of demographic characteristics. Participants in the Cogmed WMT group were significantly younger than those in the stimulant medication and combined treatment groups [mean ages being 116.77 months, *SD* = 20.39 vs. 138.20, *SD* = 35.32 and 137.20, *SD* = 29.93 months; *F*(2,87) = 5.71, *p* < .01]. Further, level of interference associated with ADHD (as rated by parents on the ADIS) was higher in the psychostimulant medication and combined treatment groups as compared to the Cogmed WMT group [means being 6.35, *SD* = 0.98 and 5.98, *SD* = 1.02 vs. 5.21, *SD* = 1.12; *F*(2,87) = 10.02, *p* < .001]. Finally, youngsters in the stimulant medication group and the combined treatment group more frequently had a low socio-economic status than youth in the Cogmed WMT group, with percentages being 65.4 and 52.2 versus 25.0%; χ^2^(1) = 9.98, *p* < .01.

Three youngsters prematurely dropped out of the study (two of them took the pre-treatment assessment but then never started with the intervention and also did not show up for the post-treatment assessment; one child initially gave permission to participate but then refused to complete the pre-treatment assessment), leaving 87 participants for the intent-to-treat analysis (33 for Cogmed WMT, 29 for stimulant medication, and 25 for the combination intervention). Nine youngsters dropped out during the intervention part of the study: five (15.1%) in the WMT condition, three (10.3%) in the medication condition, and one (4.0%) in the combined intervention condition. Within the latter condition, four participants renounced the Cogmed WMT after having started with the medication treatment and naturally “switched” to the medication condition. Thus, ultimately there were 78 participants for the completer analysis (28 for Cogmed WMT, 30 for stimulant medication, and 20 for the combination intervention). In passing, it is relevant to note that participants in the WMT conditions (i.e., Cogmed WMT and combination intervention) were only considered as completers in case they fully adhered to the Cogmed protocol and conducted all 25 sessions.

### Interventions

The Cogmed WMT was delivered to the children and adolescents at home or (in case that was not possible) at school via the official Pearson website. The program consists of visuospatial (remembering the position of objects in a grid) and verbal (remembering phonemes, letters, or digits) working memory tasks. Children had to perform these working memory exercises during 5 weeks, 45 min–1 h per day, 5 days per week. Parents (in some cases the remedial teachers at school) were instructed to supervise the children in conducting the daily set of WMT trials. The Cogmed program is developed in such way that the difficulty level of the exercises is automatically adjusted to each individual child: performance on the first trials is taken as the starting level at which the working memory span is trained and gradually improved. The Cogmed WMT was supervised by five psychologists who were officially trained and certified as Cogmed coach by Pearson Netherlands, and who followed the children’s compliance with the intervention and monitored their progress using a special log in facility which enabled them to view the files in which the results of each session conducted by the young participants were stored. In general, the program was successful in enlarging youth’s working memory capacity: the average improvement index was 27.2% (*SD* = 12.99, range 9–67; 82% of the children and adolescents reached an index ≥ 17%).

Stimulant medication was provided by four psychiatrists who were experienced in treating ADHD and other psychiatric conditions in children and adolescents. In the current study, methylphenidate—Ritalin was most often prescribed, followed by extended release variants such as Medikinet or Concerta. In most cases, psychiatrists initially started with the prescription of Ritalin, but sometimes for practical reasons (e.g., difficulties with the regular administration of the pills) or due to side effects changed to other types of medication.

The frequency/duration of the contacts with the Cogmed coaches and the psychiatrists were similar for youth in the Cogmed WMT and stimulant medication groups. There was a starting session during which (a) the psychiatrist conducted a brief medical examination and provided information about the treatment with psychostimulant medication and its procedure, while (b) the Cogmed coaches explained the rational of the WMT training and provided instructions about the use of the program and the role of the parents (or the other person who was going to assist the child in conducting the training). One week later, there was a brief follow-up session (usually by telephone) to check whether youth had started with the intervention and to address eventual questions and problems. If indicated, there were additional telephone and email contacts and occasionally even face-to-face sessions during the further course of the intervention. At the end of the training period, there was a closing session in the Cogmed WMT group during which an evaluation of the intervention took place and youth received a certificate for successfully completing the program. Around this time, children and adolescents in the stimulant medication group visited the psychiatrist for a follow-up consult. Youth in the combined treatment group had sessions and follow-up contacts with both the psychiatrist and the Cogmed coaches, and thus received twice as much care as youth in the mono-intervention groups.

### Assessment

The primary outcome measure was the *ADHD-Questionnaire* (ADHD-Q [35]), which comprises 18 items covering DSM-defined symptoms of ADHD in three domains: inattention (6 items; e.g., “has difficulties with sustaining attention in tasks or play activities”), hyperactivity (6 items; e.g., “fidgets with hands and feet or squirms in seat”), and impulsivity (6 items; e.g., “blurts out the answer before the question has been completed”). Parents, teachers, or children/adolescents themselves indicate how frequently the pertinent behavior has been present during the past 2 weeks, using a 5-point rating scale with 0 = not, 1 = now and then, 2 = regularly, 3 = often, and 4 = very often. Previous research by Scholte and Van der Ploeg [35] has shown that this rating scale is reliable in terms of internal consistency (with Cronbach’s alpha generally being well above 0.80) and temporal stability (with a 2-weeks test–retest correlation of 0.95). Further, confirmatory factor analysis has demonstrated that symptoms of inattention, hyperactivity, and impulsivity were satisfactorily represented in the scale, providing support for the construct validity of the ADHD-Q [36, 37]. Finally, scores on this questionnaire are positively associated with educational and behavioral problems, and differentiate between youth with and without an ADHD diagnosis [35, 36].

The Strengths and Difficulties Questionnaire (SDQ [38, 39]) consists of 25 items describing negative and positive attributes of children and adolescents that can be allocated to 5 subscales of 5 items each: apart from the hyperactivity-inattention problems (e.g., “restless, overactive, cannot stay still for long”, “easily distracted, concentration wanders”), there are also subscales measuring emotional symptoms (e.g., “many fears, easily scared”), conduct problems (e.g., “often loses temper”), peer problems (e.g., “rather solitary, prefers to play alone”), and prosocial behaviour (e.g., “helpful if someone is hurt, upset or feeling ill”). Parents, teachers, and youth themselves are asked to score each item on a 3-point scale with 0 = ‘not true’, 1 = ‘somewhat true’, and 2 = ‘certainly true’. Subscale scores can be computed by summing scores on relevant items (after recoding reversed items). In the present study, we employed the hyperactivity-inattention problems subscale (range 0–10) and a combined ‘other problems’ score that was based on the emotional symptoms, conduct problems, and peer problems subscales (range 0–30). The impact supplement [40] was used to enquire further about chronicity, distress, social impairment, and burden to others associated with youth’s ADHD problems. At post-treatment, the SDQ included two additional follow-up questions that specifically asked about the effects produced by the intervention: (1) did the intervention reduce the problems? and (2) did the intervention help in other ways, e.g., make the problems more bearable? Research has shown that the SDQ has adequate psychometric properties. More specifically, the internal consistency and test–retest stability of the SDQ are satisfactory [39]. Furthermore, correlations among parent, teacher, and self-report SDQ scores are moderate but compare favourably to cross-informant correlations as obtained with other psychopathology measures [39]. Evidence has also been obtained for the validity of the SDQ. That is, SDQ scores were found to correlate in the expected way with other measures of psychopathology [41] and discriminate well between youth with and without psychopathological symptoms [40]. Finally, the SDQ has proven to be an effective index for tapping ADHD symptomatology and comorbidities in clinically referred children and adolescents [42–44].

The Dutch version of the Behavior Rating Inventory of Executive Functioning (BRIEF [45]) is a 75-item parent-report questionnaire that intends to assess executive functioning in youth aged 5 to 18 years. Parents have to rate whether their child’s behavior is ‘never’, ‘sometimes’, or ‘often’ a problem. The BRIEF contains eight subscales reflecting impairments in various domains: inhibition (i.e., to control impulses and to stop engaging in a behavior), shifting (i.e., to move easily from one activity to another, to tolerate change, to switch attention), emotional control (i.e., to regulate emotional responses adequately), initiative (i.e., to start an activity and to independently generate ideas or problem solving strategies), working memory (i.e., to hold information when completing a task or performing a mental operation), planning and organization (i.e., to anticipate future events, to set goals and develop steps, to grasp the main idea), order (i.e., to put and keep order in play and living spaces), and behavior monitoring (i.e., to evaluate one’s own performance and behaviors towards other people). In the present study, we employed the BRIEF total score, a behavioral regulation index (which is composed of the subscales inhibition, shifting, and emotional control) and a meta-cognition index (which consists of the subscales initiative, working memory, planning and organization, order, and behavioral monitoring) [46]. We also considered the working memory subscale separately given its relevance as an outcome variable for the Cogmed WMT. The psychometric qualities of the BRIEF are good [45], and this appears also true in samples of children and adolescents with ADHD [47–49].

### Performance-Based Working Memory and Attentional Tests

Verbal and visuospatial working memory were measured with respectively the Digit Span test of the Wechsler Intelligence Scale for Children (WISC [33]) and the Corsi Block-tapping task [50]. During the Digit Span test, participants are verbally presented with a sequence of numerical digits, which they are asked to recall either in the normal (forward) or the reversed (backward) order. Increasingly longer sequences are tested until participants are no longer capable of correctly reproducing the sequence of the digits. In the present study, a total Digit Span score was obtained by summing the number of correctly reproduced sequences on the forward and backward trials (range 0–30). The Corsi Block-tapping task is the visuospatial equivalent of the Digit Span test. It requires participants to reproduce a sequence of movements by tapping blocks in the same (forward) or reversed (backward) order as the examiner did on a board with nine blocks placed at fixed, pseudorandom positions. The Corsi Block-tapping task also yielded a total score that ranged between 0 and 30. There is sufficient empirical support showing that performance on verbal and visuospatial span tasks indeed reflect working memory capacity [51], which appears to be significantly impaired in youth with ADHD.

The Stroop interference task [52] is a widely employed neuropsychological test that is thought to measure selective attention and response inhibition [53]. The test consists of three cards containing 100 stimuli each, which have to be processed as fast as possible. The first card requires the participants to read the names of colors that are printed in black ink. The second card asks the participants to name the color of squares that are printed in various colors. The third and final card consists of words describing colors that are printed with ink in a color that is incongruent with the word (e.g., the word ‘green’ printed in red). For each card, the response time is measured, but the interference effect is typically defined as the response time to card 3 minus the response time to card 2. A larger value is indicative for lower capacity of and thus greater problems with selective attention and response inhibition. A meta-analysis by Homack and Riccio [54] has demonstrated that children and adolescents with ADHD consistently exhibit poorer performance on the Stroop task as compared to individuals without a clinical diagnosis, although it should also be noted that youth with other psychiatric problems tend to display similar problems.

The Bourdon-Vos test [55] is a paper-and-pencil measure of sustained attention that in The Netherlands is frequently used in the neuropsychological assessment battery of children and adolescents suspect of ADHD. It is a cancellation test consisting of a single sheet on which 33 rows with 24 small patterns of three, four, or five dots. The four dots patterns are the targets that have to be cancelled in a normal reading order, as fast and as accurately as possible. A number of variables can be derived from this test. Because the experimenter measures the time needed to complete each of the 33 rows, a mean row completion time as well as a standard deviation can be obtained that are indicative for respectively the speed and the regularity of the participant’s performance. The smaller the mean row time and its accompanying standard deviation, the better a person’s ability to regulate and maintain attentional focus. In addition, omissions (i.e., missed targets), corrections (i.e., immediately noticed cancellations of non-targets), and errors (i.e., cancelled non-targets) are recorded, which together form an index of accuracy. A comprehensive psychometric evaluation is lacking, but based on the data published in the original manual [55], the Dutch evaluation committee of psychological tests (http://www.cotandocumentatie.nl) has concluded that the Bourdon-Vos test has reasonable reliability and validity.

The TEA-Ch [34] is a neuropsychological test battery for measuring youth’s attentional capacity. In this study, the following four subtests were employed: (1) the sky search subtest provides an index of selective attention (i.e., the ability to search for relevant information while ignoring irrelevant, distracting information) and consists of an A3-sheet depicting the sky above a city which is full with spaceships that fly in pairs. For 108 of the pairs the two spaceships are different, but for the 20 target pairs both spaceships are identical. Participants are instructed to search for the identical target pairs and to mark them as quickly as possible. The time needed to complete the test and the number of correctly identified target items are recorded, and eventually yield a time-per-target score. This score is corrected for motor speed by administrating a motor control version of the task during which children have to mark pairs of spaceships on a separate A3-sheet that only displays the target items. (2) the Score! Subtest measures sustained attention (i.e., the ability to preserve attention and alertness while doing a task that is not implicitly stimulating), and consists of ten trials during which children have to silently count (i.e., without the assistance of fingers) the number of tones that are produced by the computer. Each trial presents between 9 and 15 identical tones, which are separated by silent inter-stimulus intervals of variable duration (i.e., 500–5000 ms). At the end of each trial, children report the number of sounds that they have counted. This subtest yields a total score, which indicates the number of correctly counted trials (range 0–10). (3) the sky search dual task (DT) assesses divided attention (i.e., the ability to regulate, coordinate, and plan complex attention processes) and asks participants to complete a second version of the sky search subtest while simultaneously counting the number of tones produced by the computer (as was done during the Score! Subtest). The time needed to complete the test, the number of correctly identified targets and the number of correct counts are used to compute a weighted time-per-target score. (4) The Score! DT is also measuring divided attention and again consists of ten trials; this time children not only have to count the number of tones, but they also have to listen to a news bulletin (also presented by the computer) in which the name of an animal is mentioned. At the end of each trial, children have to report the number of tones they have counted as well as the animal that was mentioned in the bulletin. A total score can be derived by summing the number of correctly counted trials and the number of correctly reported animals (range 0–20). The psychometric qualities of the TEA-Ch appear satisfactory. Most of the subtests have been found to possess sufficient to good test–retest reliability [56, 57]. Furthermore, research has also provided support for the validity of the TEA-Ch, as evidenced by significant correlations with other indexes of attention (e.g., Stroop color naming task) and measures of intelligence and school performance [34, 57]. In addition, children with attention-deficit and hyperactivity disorder, who are known for their attention problems, perform significantly worse on this test as compared to other clinically referred youths [58, 59].

### Diagnostic Interview

The ADHD module of the Anxiety Disorders Interview Schedule for youth [60] was administered to parents to confirm the main clinical diagnosis of the children and adolescents. This semi-structured interview assesses the frequency and intensity of (in this case: ADHD) symptoms as well as their interference in order to check various DSM-IV based criteria [1] and to ultimately establish a diagnosable condition. There is evidence indicating that the ADIS for youth has satisfactory test–retest [61] and inter-rater reliability [62], and the concurrent validity of the ADHD module has also demonstrated to be good [63].

### Brief Follow-up Interview

One year after the post-intervention assessment had taken place, a research assistant contacted the parent(s) of the participating youth by telephone with a number of qualitative follow-up questions pertaining to (1) the current level of ADHD symptoms of the child/adolescent and the degree to which this problem interfered with daily functioning, (2) a retrospective evaluation of the intervention that the child had received for reducing attention-deficit and/or hyperactivity problems (1 = not helpful at all, 2 = somewhat helpful, 3 = helpful, 4 = extremely helpful), (3) whether the child/adolescent had continued to use the stimulant medication, and or—in case he/she had only received the Cogmed intervention—had started to use this type of medication in the past year, and (4) whether the child/adolescent had received other (additional) interventions targeting ADHD in the past year.

### Statistical Analyses

The Statistical Package for Social Sciences (SPSS Version 21) was used for calculating descriptive statistics and for performing the 3 (intervention groups: Cogmed WMT vs. stimulant medication vs. combined treatment) × 2 (assessment occasions: pre vs. post) analyses of variance (ANOVAs), with last factor being a repeated measure. The ANOVAs were conducted two times: the first time as an intent-to-treat analysis—which means that comparisons were made on the basis of the treatments that were initially chosen by parents and youth, thus regardless of eventual dropouts—and the second time as a completer analysis—which involved comparisons based on the treatments that were eventually completed. Because groups differed on a number of other variables (i.e., age, level of interference, SES, treatment confidence), ANOVAs were also conducted while correcting for these variables (ANCOVAs). In general, no significant effects of these covariates were found and therefore we decided to report the results of the uncorrected analyses. The one exception involved the analyses of the performance-based tests, which were all corrected for age. In keeping with what has been reported elsewhere in the literature [64], youth’s performance on this type of tests was found to improve significantly with increasing age. Finally, for the primary outcome measure—ADHD symptoms as measured with the ADHD-Q—effect sizes (Cohen’s *d* [65]) were computed for the pre-to-post changes observed within each of the intervention groups.

## Results

### Motives for Treatment Choice

When looking at the motives for choosing the three interventions, an interesting pattern emerged. Medication was chosen for a mix of positive and negative reasons. That is, part of the parents/youth (46.7%) selected the treatment with stimulant medication as they considered it as the intervention proven to be most effective for children and adolescents with ADHD. However, other participants chose medication because they deemed the Cogmed WMT program to be too intensive and taxing (53.3%). The choice for Cogmed WMT was mainly negatively motivated: about one-third of the participants (36.7%) preferred the training because they were strongly against the use of medication and/or feared side effects and addiction problems. Other parents/youth (40.4%) considered medication as a too rigorous step and preferred to first try Cogmed WMT. Only a minority (22.9%) chose Cogmed WMT for a positive reason, for example because they thought that this intervention would be effective. The choice for the combination intervention was without exception positively motivated (100%): all parents and youth indicated that they wanted to receive the best possible treatment, and 20% of them explicitly noted that they expected Cogmed WMT to make a significant contribution. Statistical comparisons confirmed that the combination treatment was more often chosen for a positive reason than medication [χ^2^(1) = 18.80, *p* < .001], which in turn was more frequently selected for a positive reason than Cogmed WMT [χ^2^(1) = 4.09, *p* < .05]. This imbalance in motives also partly showed itself in participants’ confidence ratings: parents and youth indicated that they expected that medication and the combination intervention (*M*s being 7.38, *SD* = 0.93 and 7.74, *SD* = 0.88, respectively) would be more helpful than Cogmed WMT (*M* = 6.63, *SD* = 1.22) [*F*(2,87) = 9.12, *p* < .001].

### Effects of Interventions on Questionnaire Data

#### Intent-to-Treat Analysis

**Fig. 2:**
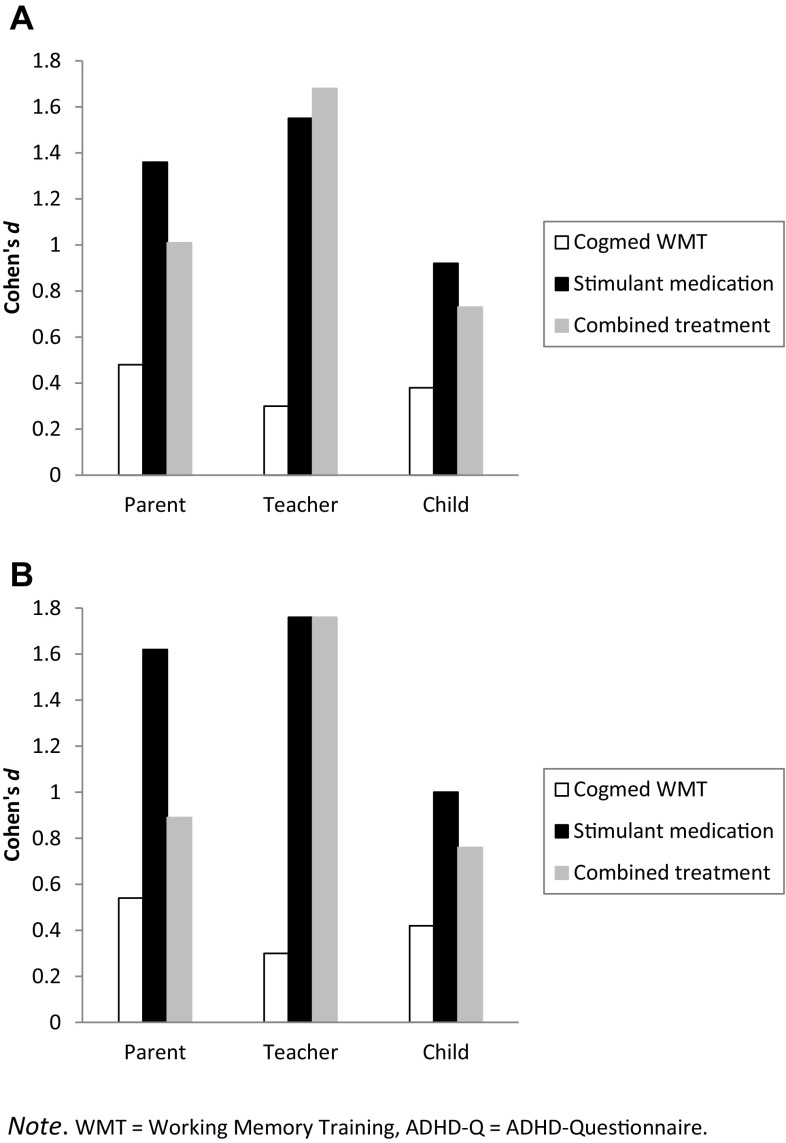
Pre- to post-treatment effect sizes in the three treatment conditions calculated for the ADHD-Q total score as obtained from parents, teachers, and youth themselves. **A** Intent-to-treat analysis and **B** completer analysis. *WMT* working memory training, *ADHD-Q* ADHD-Questionnaire

The series of 3 (groups) × 2 (occasions) ANOVAs performed on the parent and teacher data of the primary outcome measure—the ADHD-Q—revealed significant time effects, indicating a general improvement of youth’s ADHD symptomatology. More importantly, the crucial time × groups interaction effect was also significant and consistently found across all types of ADHD symptoms. As can be seen in Table 2, post-hoc comparisons revealed that according to parents and teachers reductions in ADHD symptomatology were more substantial in the stimulant medication and combined treatment groups than in the Cogmed WMT group. For the self-report version of the ADHD-Q, results were less clear-cut: the general reduction in ADHD symptoms was found for the total score as well as the three subscales, but the interaction effect was only significant for the total score and hyperactivity symptoms, with reductions in symptomatology again being larger in the stimulant medication and combined treatment groups than in the Cogmed WMT group. These findings are further illustrated in panel A of Fig. 2 in which we display pre- to post-treatment effect sizes calculated for the ADHD-Q for each of the three informants. Note that the effect sizes were considerably larger in the stimulant medication and combined treatment groups (Cohen’s *d*’s between 0.73 and 1.68, all in the medium to very large range) than in the Cogmed WMT group (Cohen’s *d*’s between 0.30 and 0.48, all in the small to medium range).

**Table 2:** Means scores (standard errors) on pre- and post-treatment questionnaires of youth in the three treatment conditions and main results of the repeated measures ANOVA (intent-to-treat analysis)

	Pre			Post			*F* Time	*F* Time × group
	Cogmed WMT (*n* = 33)	Stimulant medication (*n* = 29)	Combined treatment (*n* = 25)	Cogmed WMT (*n* = 33)	Stimulant medication (*n* = 29)	Combined treatment (*n* = 25)		
ADHD-Q P total	36.21 (2.45)^a^	45.07 (2.51)^b^	42.48 (2.70)^b^	29.46 (2.52)^c^	26.79 (2.69)^c^	27.96 (2.89)^c^	80.22***	5.77**
ADHD-Q P inattention	13.94 (0.93)^a^	16.86 (0.99)^b^	15.04 (1.07)^ab^	11.27 (1.05)^c^	10.10 (1.12)^c^	10.68 (1.20)^c^	55.96***	4.00*
ADHD-Q P hyperactivity	12.36 (1.01)^a^	14.97 (1.08)^a^	15.52 (1.16)^a^	9.85 (0.94)^b^	8.93 (1.01)^b^	10.16 (1.08)^b^	75.92***	4.46*
ADHD-Q P impulsivity	9.88 (0.97)^a^	13.38 (1.03)^b^	11.92 (1.11)^ab^	8.33 (0.88)^c^	7.83 (0.94)^c^	7.12 (1.01)^c^	50.31***	5.25**
ADHD-Q T total	38.09 (2.58)^a^	38.52 (2.75)^a^	37.24 (2.96)^a^	33.46 (2.31)^b^	18.17 (2.64)^c^	15.92 (2.65)^c^	99.87***	13.09***
ADHD-Q T inattention	13.58 (0.89)^a^	13.31 (0.95)^a^	14.08 (1.02)^a^	11.52 (0.91)^b^	6.90 (0.98)^c^	7.04 (1.05)^c^	79.74***	7.75**
ADHD-Q T hyperactivity	12.82 (1.05)^a^	14.00 (1.12)^a^	12.20 (1.21)^a^	12.49 (0.86)^a^	6.17 (0.92)^b^	4.88 (0.99)^b^	71.13***	16.84***
ADHD-Q T impulsivity	11.70 (1.05)^a^	11.21 (1.12)^a^	10.96 (1.21)^a^	9.46 (0.82)^b^	5.07 (0.87)^c^	4.00 (0.94)^c^	77.55***	6.62**
ADHD-Q C total	30.85 (2.51)^a^	38.48 (2.68)^b^	37.24 (2.88)^ab^	26.06 (2.37)^c^	23.62 (2.53)^c^	27.60 (2.72)^c^	39.64***	3.79*
ADHD-Q C inattention	10.61 (0.96)^a^	13.79 (1.03)^b^	13.40 (1.11)^b^	9.24 (0.91)^ac^	8.66 (0.97)^c^	9.84 (1.04)^c^	25.92***	2.99
ADHD-Q C hyperactivity	11.91 (0.93)^a^	14.72 (0.99)^b^	14.32 (1.07)^b^	10.61 (0.96)^a^	8.93 (1.02)^ac^	10.84 (1.10)^ac^	32.97***	4.81*
ADHD-Q C impulsivity	8.36 (0.99)^a^	10.00 (1.06)^a^	9.56 (1.14)^a^	6.42 (0.81)^b^	6.03 (0.86)^b^	6.52 (0.93)^b^	29.37***	1.23
SDQ P HI problems	6.91 (0.35)^a^	8.24 (0.38)^b^	8.04 (0.40)^b^	6.18 (0.37)^ac^	5.79 (0.40)^c^	6.36 (0.43)^c^	48.32***	4.95**
SDQ T HI problems	7.82 (0.40)^a^	8.07 (0.43)^a^	7.68 (0.46)^a^	7.27 (0.38)^a^	4.21 (0.41)^b^	4.08 (0.44)^b^	92.84***	15.94***
SDQ C HI problems	6.24 (0.34)^a^	7.38 (0.37)^b^	7.36 (0.39)^b^	5.42 (0.43)^c^	4.14 (0.46)^d^	5.20 (0.49)^cd^	64.93***	8.04**
SDQ P impact	2.24 (0.33)^a^	3.28 (0.36)^ab^	3.48 (0.38)^b^	1.61 (0.31)^ac^	0.93 (0.33)^c^	1.52 (0.35)^c^	41.54***	4.45*
SDQ T impact	1.91 (0.21)^a^	1.90 (0.22)^a^	1.96 (0.24)^a^	1.61 (0.19)^a^	0.52 (0.20)^b^	0.64 (0.22)^b^	77.50***	10.17***
SDQ C impact	1.00 (0.27)^a^	2.21 (0.29)^b^	2.00 (0.31)^b^	0.61 (0.23)^c^	0.62 (0.25)^c^	1.16 (0.26)^c^	21.23***	3.10
SDQ P other problems	5.73 (0.75)^a^	7.97 (0.79)^b^	7.44 (0.86)^ab^	5.12 (0.72)^ab^	6.72 (0.77)^b^	6.84 (0.83)^b^	4.13*	0.28
SDQ T other problems	7.70 (0.81)^a^	6.66 (0.86)^a^	7.04 (0.93)^a^	6.27 (0.70)^b^	4.17 (0.75)^b^	4.64 (0.80)^b^	29.66***	0.84
SDQ C other problems	7.85 (0.75)^a^	8.48 (0.80)^a^	7.96 (0.86)^a^	6.94 (0.71)^ab^	6.21 (0.76)^b^	6.84 (0.81)^b^	11.69**	1.06
BRIEF P total	144.49 (3.70)^a^	159.66 (3.94)^b^	156.92 (4.25)^b^	135.24 (4.16)^c^	141.07 (4.43)^c^	139.32 (4.78)^c^	38.01***	1.57
BRIEF P behavior regulation	48.15 (1.95)^a^	53.66 (2.08)^a^	53.40 (2.24)^a^	45.82 (1.80)^ab^	49.97 (1.93)^b^	47.20 (2.07)^b^	16.48***	1.24
BRIEF P metacognition	96.06 (2.41)^a^	106.14 (2.57)^b^	103.12 (2.77)^b^	89.15 (2.98)^c^	91.17 (3.18)^c^	91.92 (3.43)^c^	48.68***	2.35
BRIEF P working memory	25.03 (0.62)^a^	26.72 (0.66)^a^	25.48 (0.71)^a^	22.48 (0.81)^b^	22.14 (0.86)^b^	22.04 (0.92)^b^	61.39***	1.85

*WMT* working memory training, *ADHD-Q* ADHD-Questionnaire, *SDQ* Strengths and Difficulties Questionnaire, *HI* hyperactivity-inattention, *BRIEF* behavior rating inventory of executive functioning, *P* parent, *T* teacher, *C* child

Superscripts pertain to between-group (Cogmed WMT vs. stimulant medication vs. combined treatment on both assessment occasions separately) and within-group (pre- to post-treatment) differences; means not sharing similar superscripts differ at *p* < .05; ***p < .001; **p < .01; *p < .05

On the hyperactivity-inattention subscale of the SDQ, a similar pattern was found, but here the significant interaction effect was found in the data of all three informants. Again, youth in the simulant medication and combined treatment groups appeared to have profited more from the intervention than those in the Cogmed WMT group. The SDQ impact ratings yielded comparable results with children and adolescents in the stimulant medication and combined treatment groups demonstrating a greater reduction in the negative impact of symptoms on daily functioning as compared to those in the Cogmed WMT group, although it should also be noted that according to parents and children themselves impact ratings prior to the intervention were already (somewhat) lower in the Cogmed WMT group.

On measures of other (comorbid) symptoms as indexed by the SDQ or executive functioning problems only significant time effects were found: these symptoms and problems decreased significantly from pre- to post-treatment, but here improvements were more or less equal across the three intervention groups.

#### Completer Analysis

The 3 (groups) × 2 (occasions) ANOVAs conducted on the questionnaire data of those who had actually completed various interventions yielded a highly similar picture as the intent-to-treat analysis, which is not that surprising as relatively few participants dropped out or changed group. The crucial interaction effect of groups and occasions was found for most parent- and teacher-rated scales related to ADHD symptoms (ADHD-Q, SDQ hyperactivity-inattention subscale, SDQ impact scale), for which it was found that the reductions in symptoms were more pronounced in the stimulant medication and combined treatment groups than in the Cogmed WMT group (Table 3). As an example of this, the results on the ADHD-Q as the primary outcome variable again revealed that the effect sizes in the medication and combined treatment groups were in the medium to very large range (Cohen’s *d*’s between 0.76 and 1.76) and clearly more substantial than those documented in the Cogmed WMT group, which were all in the small to medium range (Cohen’s *d*’s between 0.30 and 0.54; see Panel B of Fig. 2). On child-report measures of ADHD symptoms and scales for assessing other (comorbid) and executive functioning problems, this pattern was less consistent.

**Table 3:** Means scores (standard errors) on pre- and post-treatment questionnaires of youth in the three treatment conditions and main results of the repeated measures ANOVA (completer analysis)

	Pre			Post			*F* Time	*F* Time × group
	Cogmed WMT (*n* = 28)	Stimulant medication (*n* = 30)	Combined treatment (*n* = 20)	Cogmed WMT (*n* = 28)	Stimulant medication (*n* = 30)	Combined treatment (*n* = 20)		
ADHD-Q P total	34.96 (2.44)^a^	44.83 (2.36)^b^	41.90 (2.89)^ab^	28.00 (2.60)^c^	24.93 (2.52)^c^	28.15 (3.08)^c^	71.75***	6.28**
ADHD-Q P inattention	13.96 (0.99)^a^	16.07 (0.95)^a^	15.20 (1.17)^a^	11.00 (1.07)^b^	9.17 (1.03)^b^	10.55 (1.27)^b^	51.20***	3.27*
ADHD-Q P hyperactivity	11.82 (1.09)^a^	15.47 (1.06)^b^	15.15 (1.30)^ab^	9.04 (0.99)^c^	8.70 (0.96)^c^	10.30 (1.17)^c^	67.97***	4.48*
ADHD-Q P impulsivity	9.14 (0.99)^a^	13.42 (0.96)^b^	11.55 (1.18)^ab^	7.96 (0.93)^ac^	7.13 (0.90)^c^	7.30 (1.10)^c^	42.69***	7.07**
ADHD-Q T total	38.36 (2.76)^a^	37.67 (2.67)^a^	38.60 (3.26)^a^	33.68 (2.42)^b^	15.90 (2.34)^c^	16.15 (2.86)^c^	95.40***	12.86***
ADHD-Q T inattention	13.64 (0.97)^a^	12.73 (0.94)^a^	14.45 (1.15)^a^	11.43 (0.95)^b^	6.03 (0.91)^c^	6.65 (1.12)^c^	78.85***	7.56**
ADHD-Q T hyperactivity	12.89 (1.09)^a^	13.93 (1.06)^a^	12.65 (1.29)^a^	12.75 (0.88)^a^	5.47 (0.85)^b^	5.25 (1.05)^b^	64.39***	16.94***
ADHD-Q T impulsivity	11.82 (1.14)^a^	11.00 (1.10)^a^	11.50 (1.34)^a^	9.50 (0.87)^b^	4.40 (0.84)^c^	4.25 (1.03)^c^	72.93***	6.22**
ADHD-Q C total	30.32 (2.70)^a^	35.87 (2.61)^a^	38.35 (3.20)^a^	24.82 (2.46)^b^	21.70 (2.37)^b^	27.95 (2.91)^b^	33.79***	2.43
ADHD-Q C inattention	10.68 (1.02)^a^	12.47 (0.99)^ab^	14.20 (1.21)^b^	8.93 (0.94)^ac^	7.77 (0.91)^c^	9.95 (1.11)^c^	24.95***	1.82
ADHD-Q C hyperactivity	11.68 (1.03)^a^	14.43 (0.99)^a^	14.00 (1.21)^a^	10.04 (1.03)^ab^	8.83 (0.99)^b^	10.55 (1.22)^b^	27.09***	3.21*
ADHD-Q C impulsivity	8.00 (1.05)^a^	9.00 (0.98)^a^	10.20 (1.24)^a^	6.11 (0.83)^ac^	5.10 (0.84)^ac^	6.95 (0.98)^c^	25.77***	1.12
SDQ P HI problems	6.79 (0.38)^a^	8.30 (0.37)^b^	8.05 (0.45)^b^	6.07 (0.41)^ac^	5.60 (0.39)^c^	6.50 (0.48)^c^	44.49***	6.20**
SDQ T HI problems	7.71 (0.43)^a^	8.03 (0.41)^a^	7.85 (0.50)^a^	7.18 (0.40)^a^	3.97 (0.39)^b^	4.05 (0.48)^b^	86.19***	15.36***
SDQ C HI problems	6.04 (0.38)^a^	7.27 (0.37)^b^	7.35 (0.45)^b^	5.11 (0.46)^c^	3.90 (0.44)^c^	5.15 (0.54)^c^	59.32***	7.21**
SDQ P impact	2.04 (0.36)^a^	3.73 (0.35)^b^	2.95 (0.42)^ab^	1.50 (0.32)^ac^	0.93 (0.31)^c^	1.30 (0.38)^c^	38.35***	6.82**
SDQ T impact	1.89 (0.22)^a^	2.07 (0.22)^a^	1.75 (0.26)^a^	1.57 (0.19)^a^	0.50 (0.18)^b^	0.45 (0.22)^b^	78.40***	11.08***
SDQ C impact	0.93 (0.30)^a^	2.17 (0.29)^b^	1.85 (0.36)^ab^	0.46 (0.23)^c^	0.60 (0.22)^c^	0.90 (0.27)^c^	21.26***	2.52
SDQ P other problems	5.46 (0.79)^a^	8.87 (0.76)^b^	6.40 (0.93)^a^	4.89 (0.74)^a^	7.40 (0.72)^b^	5.90 (0.88)^ab^	3.80	0.56
SDQ T other problems	7.29 (0.81)^a^	7.63 (0.79)^a^	6.05 (0.96)^a^	5.82 (0.70)^b^	4.40 (0.67)^b^	3.90 (0.82)^b^	34.88***	2.04
SDQ C other problems	7.39 (0.79)^a^	8.83 (0.76)^a^	7.85 (0.93)^a^	6.75 (0.79)^ab^	6.47 (0.76)^b^	6.65 (0.93)^b^	10.34**	1.51
BRIEF P total	143.50 (3.80)^a^	162.53 (3.67)^b^	152.80 (4.50)^ab^	133.11 (4.39)^c^	139.47 (4.24)^c^	137.25 (5.20)^c^	39.06***	2.28
BRIEF P behavior regulation	47.36 (2.05)^a^	55.77 (1.98)^b^	51.35 (2.42)^ab^	45.04 (1.95)^ac^	45.36 (2.05)^c^	46.00 (2.31)^c^	15.83***	0.97
BRIEF P metacognition	95.82 (2.50)^a^	106.57 (2.42)^b^	101.45 (2.96)^ab^	87.75 (3.14)^c^	89.33 (3.03)^c^	91.25 (3.71)^c^	50.20***	3.12
BRIEF P working memory	25.25 (0.63)^a^	26.67 (0.61)^a^	25.20 (0.75)^a^	22.29 (0.86)^b^	21.33 (0.83)^b^	22.10 (1.02)^b^	67.11***	3.04

Superscripts pertain to between-group (Cogmed WMT vs. stimulant medication vs. combined treatment on both assessment occasions separately) and within-group (pre- to post-treatment) differences; means not sharing similar superscripts differ at *p* < .05. ***p < .001; **p < .01; *p < .05

*WMT* working memory training, *ADHD-Q* ADHD-Questionnaire, *SDQ* Strengths and Difficulties Questionnaire, *HI* hyperactivity-inattention, *BRIEF* behavior rating inventory of executive functioning, *P* parent, *T* teacher, *C* child

### Effects of Interventions on Performance-Based Tests

#### Intent-to-Treat Analysis

The series of 3 (groups) × 2 (occasions) ANOVAs that were carried out on the performance-based tests first of all revealed significant time effects. In general, participants showed improved working memory on the WISC digit span and the Corsi block-tapping task, less interference on the Stroop test, a faster, more accurate, and more regular performance on the Bourdon-Vos test, and increased attentional capacity on some of the TEA-Ch subtests. A significant groups × occasions interaction effect was documented for the Corsi Block span task. Both the Cogmed WMT and the combined treatment group showed a significant improvement in visual-spatial working memory performance from pre- to post-treatment, which was not present in the stimulant medication group (Table 4). A groups × occasions interaction effect was also found for the Stroop test, although this result could not be substantiated by the results of the post-hoc comparisons. However, when looking at the data presented in Table 4, the decrease in the interference effect seemed to be larger in the stimulant medication and combined treatment groups than in the Cogmed WMT group, but the large variation in the scores on this test probably precluded finding more clear-cut results.

**Table 4:** Means scores (standard errors) on pre- and post-treatment performance-based tests of youth in the three treatment conditions and main results of the repeated measures ANCOVA^A^ (intent-to-treat analysis)

	Pre			Post			*F* Time	*F* Time × group
	Cogmed WMT (*n* = 33)	Stimulant medication (*n* = 29)	Combined treatment (*n* = 25)	Cogmed WMT (*n* = 33)	Stimulant medication (*n* = 29)	Combined treatment (*n* = 25)		
WISC digit span	12.42 (0.44)^a^	11.17 (0.46)^a^	12.25 (0.49)^a^	13.79 (0.51)^b^	11.62 (0.53)^ab^	13.64 (0.57)^b^	3.99*	1.59
Corsi block-tapping	13.87 (0.41)^a^	12.93 (0.43)^a^	13.37 (0.46)^a^	16.70 (0.53)^b^	13.93 (0.56)^c^	16.88 (0.60)^b^	7.16**	6.32**
Stroop interference	63.46 (6.85)^a^	85.60 (7.13)^a^	73.22 (7.68)^a^	57.90 (4.37)^b^	54.10 (4.55)^b^	51.38 (4.90)^b^	23.51***	4.78*
Bourdon-vos speed	17.25 (0.73)^a^	17.78 (0.77)^a^	17.39 (0.82)^a^	15.06 (0.51)^b^	14.49 (0.53)^b^	14.91 (0.57)^b^	35.61***	1.54
Bourdon-vos regularity	3.76 (0.31)^a^	3.66 (0.33)^a^	3.20 (0.35)^a^	2.64 (0.18)^b^	2.28 (0.18)^b^	2.16 (0.20)^b^	24.15***	0.39
Bourdon-vos accuracy	22.49 (3.84)^a^	20.62 (4.00)^a^	22.20 (4.31)^a^	15.93 (2.99)^b^	12.64 (3.12)^b^	10.42 (3.35)^b^	4.85*	1.04
TEA-Ch sky search—selective attention	4.53 (0.21)^a^	4.24 (0.22)^a^	4.32 (0.23)^a^	3.68 (0.20)^b^	3.35 (0.21)^b^	3.35 (0.22)^b^	11.98***	0.05
TEA-Ch score!—sustained attention	7.34 (0.36)	6.94 (0.38)	7.35 (0.41)	7.96 (0.36)	7.86 (0.37)	7.86 (0.40)	2.44	0.24
TEA-Ch sky search DT—divided attention	2.23 (0.90)	2.86 (0.94)	3.81 (1.01)	1.67 (0.80)	1.09 (0.84)	3.00 (0.90)	0.03	0.29
TEA-Ch score! DT—divided attention	14.31 (0.48)^a^	13.55 (0.50)^a^	14.28 (0.54)^a^	15.15 (0.49)^b^	15.88 (0.51)^b^	15.46 (0.55)^ab^	6.49*	2.11

Superscripts pertain to between-group (Cogmed WMT vs. stimulant medication vs. combined treatment on both assessment occasions separately) and within-group (pre- to post-treatment) differences; means not sharing similar superscripts differ at *p* < .05

*WMT* working memory training, *WISC* Wechsler Intelligence Scale for Children, *TEA-Ch* test of everyday attention for children, *DT* dual task

***p < .001; **p < .01; *p < .05

^A^All analyses were controlled for age

#### Completer Analysis

The 3 (groups) × 2 (occasions) ANOVAs performed on the performance-based tests of the completers yielded a comparable pattern of results. Besides the significant time effect that was found for most of these outcome variables, significant interaction effects of group × occasions were found for both the WISC Digit span and the Corsi Block span tests. Post-hoc comparisons revealed that auditory as well as visual-spatial working memory significantly improved in the Cogmed WMT and the combined treatment groups, whereas it did not in the stimulant medication group (Table 5). A significant interaction of groups and occasions was also found for Stroop interference, but again this effect could not be substantiated by follow-up tests.

**Table 5:** Means scores (standard errors) on pre- and post-treatment performance-based tests of youth in the three treatment conditions and main results of the repeated measures ANCOVA^A^ (completer analysis)

	Pre			Post			*F* Time	*F* Time × group
	Cogmed WMT (*n* = 28)	Stimulant medication (*n* = 30)	Combined treatment (*n* = 20)	Cogmed WMT (*n* = 28)	Stimulant medication (*n* = 30)	Combined treatment (*n* = 20)		
WISC digit span	12.25 (0.49)^a^	11.56 (0.46)^a^	11.92 (0.56)^a^	13.65 (0.56)^b^	11.84 (0.53)^a^	13.83 (0.65)^b^	3.75*	3.48*
Corsi block span	13.86 (0.46)^a^	12.79 (0.44)^a^	13.50 (0.53)^a^	17.11 (0.57)^b^	14.13 (0.54)^c^	17.35 (0.66)^b^	6.40*	6.04**
Stroop interference	68.68 (7.27)^a^	87.58 (6.89)^a^	71.18 (8.44)^a^	59.93 (4.90)^b^	54.07 (4.64)^b^	52.06 (5.69)^b^	26.92***	4.21*
Bourdon-vos speed	17.20 (0.82)^a^	18.35 (0.78)^a^	16.78 (0.95)^a^	14.80 (0.56)^b^	14.75 (0.53)^b^	14.77 (0.65)^b^	28.66***	2.95
Bourdon-vos regularity	3.76 (0.35)^a^	3.71 (0.33)^a^	3.22 (0.40)^a^	2.57 (0.19)^b^	2.25 (0.18)^b^	2.08 (0.22)^b^	19.21***	0.16
Bourdon-vos accuracy	22.84 (4.20)^a^	20.00 (3.98)^a^	26.13 (4.88)^a^	15.22 (3.25)^b^	12.95 (3.08)^b^	11.27 (3.78)^b^	4.92*	2.19
TEA-Ch sky search—selective attention	4.52 (0.23)^a^	4.30 (0.22)^a^	4.34 (0.27)^a^	3.52 (0.21)^b^	3.41 (0.20)^b^	3.35 (0.25)^b^	12.46**	0.07
TEA-Ch score!—sustained attention	7.38 (0.41)	7.02 (0.39)	7.25 (0.48)	8.10 (0.38)	7.81 (0.36)	8.09 (0.44)	3.40	0.01
TEA-Ch sky search DT—divided attention	2.38 (1.01)	2.79 (0.95)	3.90 (1.17)	1.91 (0.92)	2.12 (0.87)	1.83 (1.07)	0.05	0.39
TEA-Ch score! DT—divided attention	14.18 (0.53)^a^	13.68 (0.50)^a^	14.23 (0.61)^a^	15.19 (0.54)^b^	15.83 (0.51)^b^	15.39 (0.62)^ab^	7.23**	1.22

Superscripts pertain to between-group (Cogmed WMT vs. stimulant medication vs. combined treatment on both assessment occasions separately) and within-group (pre- to post-treatment) differences; means not sharing similar superscripts differ at *p* < .05

*WMT* working memory training, *WISC* Wechsler Intelligence Scale for Children, *TEA-Ch* test of everyday attention for children, *DT* dual task

*** *p* < .001, ** *p* < .01, * *p* < .05, † *p* = .05

^A^All analyses were controlled for age

### Subjective Evaluation of Interventions at Post-treatment and Follow-up

The post-treatment SDQ included some questions evaluating the received intervention. As can be seen in Table 6, the stimulant medication intervention was generally evaluated as the best intervention: that is, between 50.0 and 80.0% of the parents, teachers, and children considered this treatment effective in reducing ADHD symptoms and/or making the problem more bearable. The combined treatment closely followed as second best with somewhat lower percentages between 35.0 and 65.0%, while the Cogmed WMT was ranked third with percentages between 3.6 and 57.1%.

**Table 6:** Percentages of youth in each of the three treatment groups for whom the intervention was evaluated as positive^A^ on the post-treatment SDQ and the follow-up interview

	Cogmed WMT (*n* = 28)	Stimulant medication (*n* = 30)	Combined treatment (*n* = 20)	χ^2^(2)
Post-treatment				
SDQ P reduced ADHD symptoms	14.3^a^	50.0^b^	35.0^ab^	8.35*
SDQ T reduced ADHD symptoms	3.6^a^	66.7^b^	55.0^b^	26.00***
SDQ C reduced ADHD symptoms	21.4^a^	60.0^b^	60.0^b^	10.75**
SDQ P made problem more bearable	35.7^a^	80.0^b^	55.0^ab^	11.72**
SDQ T made problem more bearable	17.9^a^	60.0^b^	60.0^b^	12.89**
SDQ C made problem more bearable	57.1	76.7	65.0	2.52
One-year follow-up interview (P)				
Improved ADHD symptoms/daily functioning	7.1^a^	53.3^b^	65.0^b^	20.07***
Helpfulness of intervention	10.7^a^	76.7^b^	90.0^b^	37.96***

Percentages not sharing similar superscripts differ at *p* < .05

*WMT* working memory training, *SDQ* Strengths and Difficulties Questionnaire, *P* parent, *T* teacher, *C* child

^A^All variables were dichotomized so that chi square tests could be conducted without violating the ‘frequencies should be greater than 5’ assumption

The results of the 1-year follow-up interview revealed that parents indicated that stimulant medication and the combined treatment had been more helpful and more effective in reducing ADHD symptoms and improving youth’s daily functioning than the Cogmed WMT. Percentages of youth taking Ritalin or some other stimulant agent were 86.7% in the stimulant medication group and 95.0% in the combined treatment group, indicating that the majority of youth had continued this treatment. About one-third of the youth (31.0%) had started with a new intervention during the past year. Interestingly, most of them were from the Cogmed WMT group of which almost half of the children and adolescents (46.4%) had started with the use of stimulant medication. Examples of other interventions were the use of homeopathic medication (e.g., fish oil), placement in a special school, semi-residential care, and participation in social skills training.

## Discussion

This naturalistic study examined treatment preferences for Cogmed WMT versus psychostimulant medication in youth diagnosed with ADHD as well as the clinical effectiveness of these interventions. With regard to the treatment preferences, it can be concluded that Cogmed WMT is a viable treatment option for youth with ADHD and their parents: for 38.9% of the participants in this study Cogmed WMT was selected as the stand-alone intervention, whereas a further 27.8% chose WMT in combination with psychostimulant medication. As no other treatment alternatives (e.g., neurofeedback, behavior therapy, elimination diet) were offered to our participants, the results remain silent about the ‘real’ preference for Cogmed WMT, but at least these findings indicate that a substantial proportion of youth and parents is open to this non-pharmacological treatment of ADHD. Admittedly, when looking at the motives for choosing various interventions, it must also be concluded that Cogmed WMT was frequently chosen for a negative reason: that is, more than three quarters of the participants selected this intervention because they were strongly against the use of medication, feared side-effects or addiction problems, or considered medication as a too rigorous step. This nicely echoes the literature indicating that parents often dismiss psychostimulant medication as a treatment for young people with ADHD [16] and that this deposition is often based on misconceptions and faulty negative beliefs about this type of intervention [19].

Treatment with stimulant medication was chosen for a mix of positive and negative reasons. Nearly half of the participants selected this intervention because it was considered to be most effective for youth with ADHD. The other participants chose medication because they thought that the Cogmed WMT (1 h per day, 5 days per week, 5 weeks long) would be too intensive and hence too taxing for them to conduct. Indeed some young participants in the Cogmed group reported that the training was taxing and for their parents it was quite difficult to keep these children motivated to perform the (almost) daily exercises. For this reason, researchers have begun to incorporate game elements in WMT to improve the motivation and engagement of youth [66]. Meanwhile, there were also children and adolescents in the present study who were really enthusiastic and really liked the Cogmed computer program. In addition, the overall compliance with the training was fairly high and the mean improvement index (27.2, with 82% reaching an improvement index of at least 17%) found in this study comes close to what has been reported in the Cogmed literature. Finally, the drop-out rate in the WMT group was fairly low (i.e., 15.1%). So the overall conclusion seems to be that Cogmed WMT was an acceptable and feasible intervention for this clinically referred population of youth with ADHD.

When looking at the effects produced by the interventions on our primary outcome measure, especially the parent and teacher version of the ADHD-Q, it can first of all be concluded that there was little difference between the results of the intent-to-treat and the completer analyses (probably as a result of the fairly low drop-out rates). In general, all children and adolescents showed significant improvement in ADHD symptomatology from the pre- to post-assessment. This means that in general youth in all three groups displayed lower levels of inattention, hyperactivity, and impulsivity following treatment. It was also noted that reductions in ADHD symptomatology were consistently larger in the stimulant medication and the combined treatment groups than in the Cogmed WMT group. More precisely, the effect sizes found for stimulant medication and combined treatment were all in the medium to very large range, whereas those obtained for Cogmed WMT were in the small to medium range. Admittedly, previous research has yielded variable effect sizes for these interventions in youth with ADHD, probably as a result of variations in population, outcome measures used, type of informant, and study design (i.e., blinded or not). However, the results are well in line with the general conclusion of meta-analytic studies indicating that the effects on ADHD symptoms produced by stimulant medication are considerably larger than those produced by cognitive training programs such as Cogmed WMT [29, 67].

Analysis of the data obtained with the self-report version of the ADHD-Q confirmed the general picture that all children and adolescents to some extent profited from the interventions. More precisely, youth themselves also reported to experience reduced levels of ADHD symptomatology following treatment with psychostimulant medication, Cogmed WMT, or the combined intervention. The interaction effects of treatment groups and time (indicating the differential effectiveness of interventions) were less robust (and in the case of the completer analysis even mostly non-significant) when the child-report ADHD-Q was employed as outcome measure. The main reason for this was that—according to youth themselves—reductions of their ADHD symptoms following psychostimulant medication and the combined treatment were smaller than the reductions of these symptoms as observed by parents and teachers. On the one hand, this may be due to youth underreporting these externalizing symptoms [68], but on the other hand it is also possible that youth have a different (i.e., less positive) perspective on the intensity of symptoms, associated impairments, and overall functioning [69].

The hyperactivity-inattention subscale and the impact supplement of the SDQ generally yielded the same results as those obtained with the ADHD-Q. Thus, these measures also indicated that symptoms of ADHD and their negative impact on youth’s daily functioning decreased from the pre- to post-treatment assessment. In addition, children and adolescents in the stimulant medication and combined treatment groups appeared to profit more from their intervention than youth receiving the Cogmed WMT, and this was most consistently the case when the SDQ hyperactivity-inattention subscale and the impact supplement were completed by the parent and the teacher. For SDQ other problems and BRIEF executive functioning significant time effects were found, indicating that co-occurring psychopathological symptoms and problems with executive functions related to behavior regulation, meta-cognition, and working memory generally were less prominent following all interventions. No significant interaction effects emerged, implying that all treatments were equally effective in reducing comorbid symptoms and improving executive functioning.

The analyses conducted on the neuropsychological scores revealed that youth showed improved performance on seven (completer analysis) to eight (intent-to-treat analysis) out of ten tests. More precisely, following the interventions, youth on average remembered more numbers on the WISC digit span, reproduced a larger sequence of tapping movements on the Corsi Block span, showed less interference on the Stroop color-naming task, were faster and more accurate on the Bourdon-Vos test, and displayed improved selective and divided attention on a number of TEA-Ch tasks. For a number of variables, the interaction of groups by time was also significant. To begin with, it was found that youth in the Cogmed WMT and the combination treatment groups showed a larger improvement in their performance on the WISC digit span (only completer analysis) and the Corsi Block span test (intent-to-treat and completer analysis) as compared to youth in the stimulant medication group. Thus, the interventions that included the WMT component appeared to produce a (somewhat) better effect on indices of verbal and visuospatial working memory, which is in agreement with was has been reported in previous studies [27, 70–75] and of course not that surprising because improvement of working memory capacity is the primary goal of this type of training [24]. In relation to this, various scholars have questioned the way that working memory improvement has been evaluated in Cogmed research; they stress the importance of measuring training effects by means of valid working memory tasks that less closely resemble the method of training [28, 76]. Further, a significant interaction effect was also found on the Stroop color-naming test. Inspection of the interference scores revealed that the stimulant medication and the combined treatment groups displayed greater improvement on this measure than the Cogmed WMT group, suggesting that youth receiving an intervention including medication fared better on this outcome measure. However, it should also be noted that this impression could not be substantiated by the post-hoc tests, probably as a result of the large variation in scores.

The subjective evaluations obtained from the three informants at post-treatment were largely in keeping with the data as obtained from our quantitative assessments: that is, in general parents, teachers, and youth themselves evaluated the stimulant medication and combined treatment as considerably more helpful in reducing ADHD symptomatology and making the problem more bearable than Cogmed WMT. At the 1-year follow-up interview with the parents, this picture was unchanged, although it should be admitted that this comparison might have been influenced by the fact that for most youth medication treatment was continued after the post-treatment assessment, whereas the Cogmed training had stopped at that time. The subjective evaluations confirm previous reports indicating that the use of stimulant medication is clinically effective and significantly improves quality of life and functional outcomes in a substantial proportion of the children and adolescents with ADHD [77], and that Cogmed WMT, just like other non-pharmacological interventions (e.g., neurofeedback [78]), is clearly less persuasive in this regard [79]. Meanwhile, this does not preclude the possibility that Cogmed WMT may have an important function in daily clinical practice. As also noted in the present study, a substantial proportion of the parents refuses stimulant medication as a treatment option for their child with ADHD, and for these parents Cogmed WMT might be a viable alternative. By supervising their own child during the training, parents may acquire more insight in the problem of their child (e.g., observe how difficult it is for their offspring to concentrate on a cognitively challenging task), which eventually paves the way for them to accept pharmacotherapy as an intervention. Indeed, almost half of the parents in the WMT group (46.4%) who had been refusing or reluctant to accept medication as a treatment option for their child at the beginning of this study, changed their mind and accepted this type of treatment within one year following the Cogmed intervention.

It should be emphasized that this study was not a randomized controlled trial and that it is not appropriate to use these data to directly compare the effects of Cogmed WMT, stimulant medication, or the combined treatment. For example, because we allowed parents and youth to choose their preferred intervention, the three treatment groups were not fully comparable with regard to levels of symptomatology and problems. That is, according to parents and youth themselves, the participants in the Cogmed WMT group displayed lower (although still clinically elevated) levels of ADHD symptoms, associated interference, comorbid symptoms, and executive functioning at pre-treatment than the participants in the stimulant medication and combined treatment groups. Further, the aforementioned imbalance in motives also accounted for differences in confidence ratings across the three groups, with parents and youth expecting that medication and the combination intervention would be more helpful than Cogmed WMT. On the basis of these inequalities, it is better to view the present study as a naturalistic trial testing the feasibility and effects of Cogmed WMT in common clinical practice, and delimitate its role besides pharmacological interventions for youth with ADHD. Further, when evaluating the treatment effects obtained in this study, one should bear in mind that alongside the described interventions (i.e., Cogmed WMT, stimulant medication, combined treatment) all young participants and their parents received psychoeducation, which is generally seen as the first step in the standard care for children and adolescents with ADHD [9, 10]. Thus, the quite robust general treatment effects observed in this study may be at least in part due to a better understanding and handling of ADHD as a result of this common treatment component [80].

## Summary

ADHD is a debilitating neurodevelopmental disorder causing significant problems in youth’s daily functioning. While there is no curative intervention for ADHD, good care and treatment may help to normalize the behavior of these youngsters and to improve their eventual prognosis. Pharmacotherapy with psychostimulant medication is currently considered as the best treatment option, and the current data confirm this by demonstrating the robust clinical effectiveness of this intervention. However, it is also a fact that many youth and their parents are not eager to adopt this type of treatment and this was the case in our clinical practice where we presented youth with ADHD and their parents with a ‘menu’ of treatment including Cogmed WMT, stimulant medication, and the combination of these interventions. About one-third of the youth with ADHD and their parents chose the non-pharmacological treatment of Cogmed WMT, and the vast majority of them (77.1%) did so because they were either strongly against the use of medication, feared side effects and addiction problems, or considered it as a too rigorous step. Interestingly, it was found that a considerable number of the youth and parents who had initially selected the Cogmed WMT changed their opinion in the year following the intervention and were willing to accept medication treatment. On the one hand, this change may have been due to the fact that the clinical effects produced by Cogmed WMT were quite modest and so for most youth there was still an urge for additional treatment. On the other hand, we also observed that the WMT made parents more aware of the specific problems of their offspring. That is, while conducting the program together with their child, they seemed to get a better picture of its attention problems, hyperactivity, and impulsivity under ‘scholastic’ conditions. Thus, rather than turning Cogmed WMT down as a rather ineffective intervention for youth ADHD, we would like to emphasize that this intervention may have an appropriate role in clinical practice.
